# Development and evaluation of a school-based bullying prevention program (*Bullying&You*): study protocol for a cluster randomized trial

**DOI:** 10.1186/s13063-025-08821-x

**Published:** 2025-03-31

**Authors:** Vanessa Jantzer, Franziska Neumayer, Stefan Lerch, Michael Kaess

**Affiliations:** 1 Department of Child and Adolescent Psychiatry, Center for Psychosocial Medicine, University Hospital Heidelberg, Blumenstrasse 8, 69115 Heidelberg, Germany; 2 Faculty of Behavioral and Cultural Studies, Institute of Psychology, Heidelberg University, Hauptstrasse 47-51, 69117 Heidelberg, Germany; 3 University Hospital of Child and Adolescent Psychiatry and Psychotherapy, University of Bern, Stöckli, Bolligenstrasse 111, 3000 Bern, Switzerland

**Keywords:** Bullying, Mental health, School, Prevention, Adolescents, Cluster randomized trial

## Abstract

**Background:**

Bullying victimization affects one in ten schoolchildren in Europe and has far-reaching negative consequences for mental health and school achievement. Although school-based bullying prevention programs seem overall capable of reducing the frequency of bullying, the continuous development, improvement, and rigorous evaluation of bullying prevention programs with enhanced feasibility and efficacy is critical. Consequently, we developed the program *Bullying&You*, which applies a blended-intervention approach to school-based bullying prevention based on latest empirical knowledge regarding effective program components and program-related facilitators. We aim to test its efficacy within a cluster randomized trial (CRT).

**Methods:**

*Bullying&You* will be implemented and evaluated in 40 schools (estimated total *n* = 8500 pupils) in Germany. The effectiveness of the program will be investigated in a CRT comparing 20 schools in the intervention group (IG; starting immediately with the program) with 20 schools in the waiting control group (CG; starting with a 1-year delay). The target group of the program are pupils in grades 3–9, as well as the whole school staff. All pupils will be asked to complete questionnaires concerning their bullying experiences (as victims, perpetrators, and bystanders) and mental health at baseline (T0) and two annual follow-ups (T1 and T2). The main endpoint of the trial is the reduction of bullying (prevalence of victims and perpetrators of direct, indirect, and cyberbullying) at 1-year follow-up (T1) in the IG compared to the CG. Secondary endpoints are psychopathology and self-harm behaviour. In addition, further research questions include (a) which specific components of the program prove to be most effective and (b) whether there are certain characteristics that predict program success at the individual level.

**Discussion:**

School-based bullying prevention programs still lack rigorous evidence for their efficacy. In addition, dissemination of bullying prevention programs has previously been hampered by the high need of resources required from schools for their implementation. The program’s blended-intervention approach allows for a time-efficient and flexible implementation, while the continuous monitoring of the progress ensures program fidelity and strengthens adherence. If proven effective, *Bullying&You* has the potential to contribute to filling the gap in systematic dissemination of bullying prevention among youth.

**Trial registration:**

German Clinical Trials Register DRKS00028183. Registered on 02 March 2022.

## Administrative information

Note: the numbers in curly brackets in this protocol refer to SPIRIT checklist item numbers. The order of the items has been modified to group similar items (see http://www.equator-network.org/reporting-guidelines/spirit-2013-statement-defining-standard-protocol-items-for-clinical-trials/).

**Table:** 

Title {1}	Development and evaluation of a school-based bullying prevention program (*Bullying&You*): study protocol for a cluster randomized trial
Trial registration {2a and 2b}	DRKS00028183, German Clinical Trials Register, https://drks.de, registered on 2 March 2022
Protocol version {3}	Version 1.1
Funding {4}	This research is funded by the foundation of Baden-Wuerttemberg (*Baden-Wuerttemberg Stiftung*).
Author details {5a}	1 Department of Child and Adolescent Psychiatry, Center for Psychosocial Medicine, University Hospital Heidelberg, Blumenstrasse 8, 69,115 Heidelberg, Germany 2 Faculty of Behavioral and Cultural Studies, Institute of Psychology, University of Heidelberg, Hauptstrasse 47–51, 69,117 Heidelberg, Germany 3 University Hospital of Child and Adolescent Psychiatry and Psychotherapy, University of Bern, Stöckli, Bolligenstrasse 111, 3000 Bern 60, Switzerland
Name and contact information for the trial sponsor {5b}	University Hospital Heidelberg, Im Neuenheimer Feld 325, 69120 Heidelberg, Germany
Role of sponsor {5c}	The sponsor and funders do not have any authority over research activities.

## Introduction

### Background and rationale {6a}

#### The need for bullying prevention

Bullying is one of the most common forms of youth violence and is by now acknowledged as a serious public health concern, affecting children and adolescents in all parts of the world [1]. According to the Centers for Disease Control and Prevention, bullying among youths is ‘any unwanted aggressive behaviour(s) by another youth or group of youths who are not siblings or current dating partners that involves an observed or perceived power imbalance and is repeated multiple times or is highly likely to be repeated. Bullying may inflict harm or distress on the targeted youth including physical, psychological, social, or educational harm’ ( [2], p. 17). The 2017/18 cycle of the Health Behavior in School-aged Children (HBSC) study outlined that bullying victimization affects one in ten school children in Europe and Canada [3]. For Germany, this rate was found to be similarly high (8.3%; [4]).

Bullying among adolescents is associated with far-reaching consequences for the mental health of both the victims and the perpetrators [5]. The impact of bullying for the development of mental disorders is highlighted by a recent meta-analysis [6] which showed evidence for a direct path from peer victimization to poor mental health outcomes including depression, non-suicidal self-injury, and suicide attempts. Furthermore, longitudinal studies suggest that victims of bullying are more likely to show problematic Internet use and gaming behaviour [7, 8]. A recent study from our research group also revealed an explicit increase in psychopathology within 1 year as a result of developing victimization [9]. Correspondingly, an improvement of mental health after the termination of bullying was observed. Moreover, bullying victimization is negatively related to cognitive-motivational factors, which, in turn, are associated with poorer academic achievement [10]. These outcomes highlight the notion that bullying among young people is a public health problem, and effective intervention and prevention programs are needed to reduce this problem.

Simultaneously, the Lancet Psychiatry Commission on youth mental health concludes that we are in the midst of a mental health crisis in children and adolescents [11]. There is evidence that the mental health of young people worldwide has deteriorated over the last two decades, and the incidence of mental illness has increased due to global crises and negative changes in many societies. Mental illnesses have a peak age of onset of 15 years and are the main reason for premature death from physical illness or suicide. One of the Commission’s key demands, therefore, is the expansion of prevention strategies for children and young people. For this purpose, the school setting offers decisive advantages over other contexts: accessibility (existing compulsory education), economy (available human and material resources), and conformity with the educational mandate (promotion of mental health can be understood as part of educational work) [12]. Bullying prevention is health promotion and, especially since bullying mainly takes place in the school context, it is essential to start there. Unfortunately, the ‘Green List Prevention’ (an online database of recommended German-language prevention programs in the areas of family, school, children/youth, and neighbourhood) states that although there is a wealth of preventive programs in Germany, there are only a few high-quality evaluation studies that show which programs are actually effective [13]. Currently, only two school-based programs on bullying that have proven effectiveness are listed there, and only one of these two programs addresses both online and offline bullying. However, this program is only suitable for secondary school students and is strongly regionally limited to the Berlin area, with only a few schools from the state of Baden-Wuerttemberg taking part.

#### The challenges in bullying prevention

A recent meta-analysis including 100 independent evaluations of anti-bullying programs found that, overall, school-based programs were effective in reducing bullying perpetration and victimization [14]. The overall effect reported was a relative reduction of bullying between 15 and 20%, with wide variation in effect sizes between the single programs and studies [15]. While this is encouraging overall, there are several unresolved issues that need to be targeted by future developments of bullying prevention programs.

Another meta-analysis [16] revealed that the presence of two main intervention components was significantly correlated with effective reduction in school-bullying victimization, as well as perpetration outcomes: informal peer involvement and information for parents. Informal peer involvement refers to the use of in-class or group-based discussion, while information for parents was defined as providing parents with information about bullying-related issues through take-home letters or leaflets. Both components can only be covered in whole-school approaches which actively involve all actors within the school environment in anti-bullying activities. Thus, even if whole-school approaches are often related to more effort, they can most likely achieve far-reaching effects. In contrast, low-threshold programs are often not designed to be long-lasting and sustainable, so they do not result in any permanent changes in the school culture and bullying issues.

Many existing programs—in particular those applying whole-school approaches—are costly both in monetary terms as well as in expenditure of time, requiring high levels of training and staff commitment that is often reduced if programs are not tailored to the needs of the school staff [16, 17]. This results in a high threshold for the implementation of these programs. In line with this observation, our own evaluation of the Olweus Bullying Prevention Program (OBPP) in Germany—one of the most effective bullying prevention programs worldwide—resulted in a satisfactory bullying reduction of 25% after 2 years. However, enthusiasm was clearly diminished by the extremely low participation rate of schools during the recruitment and advertising phase (1.9%; [18]).

In conclusion, broad and effective bullying prevention require programs that (i) target the whole school with empirically derived components in order to be effective, (ii) include the school staff’s perspective in the development of the program, and (iii) support time- and cost-efficient as well as flexible implementation and maintenance of these prevention components in order to increase feasibility and subsequent dissemination.

#### The program Bullying&You

To address these challenges, our group developed the school-based bullying prevention program *Bullying&You* (in German: *Mobbing&Du*). It is designed as a whole-school approach including multiple levels of the socio-ecological model, since this approach has been shown to achieve the greatest effects [16]. The program involves both universal actions targeted at all pupils and teachers, as well as indicated actions targeted at pupils involved in a bullying episode. It is based on latest empirical knowledge regarding effective program components and program-related facilitators. According to the current evidence, a variety of components are significantly associated with larger mean effect sizes, including a clear anti-bullying policy, classroom rules, identification of hot spots in school, curriculum materials for teachers and pupils, use of video material and teacher trainings, information for parents, informal peer involvement (i.e. whole-class or small group discussions), work with victims, and non-punitive disciplinary methods [14, 19, 20]. Therefore, all of the aforementioned components are included as part of *Bullying&You*.

In addition to searching for components that were found to be effective in previous research, we consulted a focus group with experts from the field of bullying prevention. This is one important step in the development and implementation of prevention programs. For instance, Herkama et al. [21] identified in focus group interviews the following program-related facilitators for the sustainment of the KiVa anti-bullying program: systematic program structure, clear guidelines on how to address acute cases of bullying, user-friendly materials, program adaptability, information about bullying as a phenomenon, support from program developers, as well as realistic expectations. We considered these findings in addition to the central findings of our own focus group which were (i) teacher role plays in small groups are needed to practise on-the-spot intervention; (ii) guidelines for the conversations with those involved in bullying to help teachers to act confidently; (iii) a practical tool for documenting observed bullying and passing on information would be helpful; (iv) a core team of teachers/social workers from each school needs training and continuous support to build up the program, to motivate the participants, and to monitor the progress; and (v) regular pupil surveys are needed to identify the school-specific challenges and to monitor the progress in dealing with these needs.

*Bullying&You* follows a blended-intervention approach combining analogue as well as digital components. According to Nocentini et al. [22], Information and Communication Technologies (ICTs) constitute suitable tools for bullying intervention and prevention. Computer-delivered interventions have several advantages compared to traditional methods: (i) they make information selectively available to the user at a time, speed, and format of presentation controlled by the user; (ii) they allow multimodal and multisensory experiences; (iii) they are attractive to children and adolescents; (iv) they allow pupils to practise skills or review information as often as they wish [22]. Aside from these advantages, online methods are cost-effective and practical due to their flexibility [14]. Considering the enormous development of digital tools and their importance for adolescents, ICTs are still under-used in the field of bullying prevention. This is shown in a review analysing the use of ICTs for the prevention and intervention against bullying and cyberbullying, where only 13 programs could be included. From these 13 programs, only 2 programs (NoTrap! by Menesini et al. [23] and KiVa by Salmivalli et al. [24]) had preventive as well as interventional components, combined face-to-face and ICTs-mediated content, addressed online as well as offline bullying, and showed some evidence of effectiveness [22]. Similarly, a more recent meta-analysis on the effectiveness of digital health interventions (DHIs) in reducing bullying and cyberbullying including 16 studies highlights the promising effects of digital health approaches in anti-bullying work and the need for the development of more effective DHIs [25]. Thus, *Bullying&You* clearly fills a gap in anti-bullying work by using digital components.

Overall, *Bullying&You* is based on empirical knowledge on effective strategies in the field of school-based bullying prevention, which were supplemented by recommendations from our own focus group. Moreover, the program was developed as a blended-intervention approach including innovative online components to facilitate easy accessibility and broad utility.

#### The Bullying&You cluster randomized trial (CRT)

High-quality basic research is needed to guide innovation in prevention programs and to rigorously test prevention strategies in methodologically sound outcome evaluations. Reviews indicate that there is substantial variation in the methodological quality of outcome evaluations, making it difficult to draw firm conclusions about genuine treatment effects [26]. Randomized trials are considered to be the ‘gold standard’ of experimental evaluations. School-based prevention programs showed general effectiveness in reducing bullying perpetration and victimization in numerous randomized trials within the last two decades [14]. Still, the potential influence of anti-bullying interventions on the mental health of victims and perpetrators of bullying is largely unknown, even if a recent meta-analysis is cautiously optimistic. The inclusion of 69 randomized trials revealed that anti-bullying interventions showed statistically significant effectiveness in improving mental health problems (such as anxiety and depression) with small effect sizes [27]. Our program, which is likely more feasible, efficient, and potentially more economical through blended-intervention, aims to achieve these positive effects on the frequency of bullying and on the mental health of those affected. By investigating the effectiveness of specific program components in our CRT, we may be able to better understand which elements work best in reducing bullying perpetration and victimization. Such research would have important implications for policy and the development of future anti-bullying programs [14].

## Objectives {7}

The primary aim of this CRT is to evaluate the effectiveness of the newly developed school-based blended-intervention bullying prevention program *Bullying&You*. We aim to investigate whether *Bullying&You* leads to a reduction in victimization and perpetration rates among pupils in the intervention group (IG) compared to a waiting control group (CG) within 1 year (T1) and 2 years (T2), respectively (primary objective). Secondary aims are to explore whether the program leads to a reduction in psychological distress, self-harming behaviour, and problematic Internet use in the IG compared to the CG (second objective). In addition, exploratory analyses will aim to evaluate which specific components of the program are particularly effective in reducing bullying (third objective), and which characteristics of pupils predict program success at the individual level (fourth objective).

## Trial design {8}

In this CRT, the implementation of a blended-intervention bullying prevention program (*Bullying&You*) in schools is compared to schools on a waiting list for program implementation (1-year delay; access for teachers to psychoeducational material). This is a superiority cluster randomized trial applying a parallel group design. The allocation ratio for schools is 1:1.

## Methods: participants, interventions, and outcomes

### Study setting {9}

The study will be conducted at 30 secondary schools and 10 primary schools within the state of Baden-Wuerttemberg, Germany.

### Eligibility criteria {10}

Schools are included in the CRT if they are located in the state of Baden-Wuerttemberg and if they agreed to implement the bullying prevention program. Concurrently, primary schools are included if they have at least 200 pupils or if they belong to a secondary school that is already taking part in the program. Secondary schools are included if they have at least 50 pupils.

Pupils from these schools are included if they are in grades 3 to 4 in primary schools and grades 5 to 9 in secondary schools (girls and boys, no age restrictions). In addition, all teachers of participating schools will be invited to participate in the study.

### Who will take informed consent? {26a}

All pupils are informed about the purpose, content, and conditions of the study by members of our research team in class as well as by information leaflets. They are also informed that they can either participate in the surveys as part of the prevention program, or additionally participate in the respective study that aims to evaluate the program. At the beginning of each online survey, pupils are asked for their active online consent to participate in the study (‘Would you like to participate in the study?’—the answer option ‘no, I do not want to, or my parents do not want me to participate in the study’ directly leads to the first question of the survey; the answer option ‘yes, I would like to participate in the study’ leads to the request to create an individual code). In the former case, anonymized data will only be used as feedback for the respective school as part of the prevention program. In the latter case, data will be used for research purposes including the CRT.

Their respective caregivers also are informed by information leaflets, and all are given the opportunity to contact our research team for questions and/or opt-out from their children’s participation in the research. As the study was approved as opt-out procedure by the Ethics Committee of the Medical Faculty at the University of Heidelberg (S-471/2020), as well as by the Ministry of Education of the state of Baden-Wuerttemberg, written informed consent was not needed by parents. The waiver for parental consent was obtained as the study is expected to result in great benefits for the target group and could hardly be conducted otherwise due to the expected low response rate. Beyond that, the study is a non-invasive low-risk study.

Teachers will be asked to participate in anonymous online surveys throughout the study and will receive written information on this. No informed consent is required.

### Additional consent provisions for collection and use of participant data and biological specimens {26b}

This trial does not include the collection of biological specimens.

## Interventions

### Explanation for the choice of comparators {6b}

The IG will immediately start with the implementation of *Bullying&You*. Since bullying is known to have a variety of harmful consequences, it would be unethical to prevent schools in the CG from receiving the anti-bullying program. Therefore, we decided to compare the IG with a waiting CG that will implement the program with a delay of 1 year. Furthermore, past research shows that it can be very difficult to recruit schools for a randomized trial design [16]. This is most likely to be successful if schools in the CG are promised to receive the same treatment as the IG but with a specified delay. In order to keep the motivation in the CG comparably high as in the IG and to bridge the waiting time, teachers in CG schools obtain psychoeducational material regarding bullying.

### Intervention description {11a}

The main goal of *Bullying&You* is to reduce the frequency of bullying. This goal was broken down into the following secondary goals:

- Identify bullying
- Stop bullying
- Prevent bullying
- Keep the program running

**Fig. 1:**
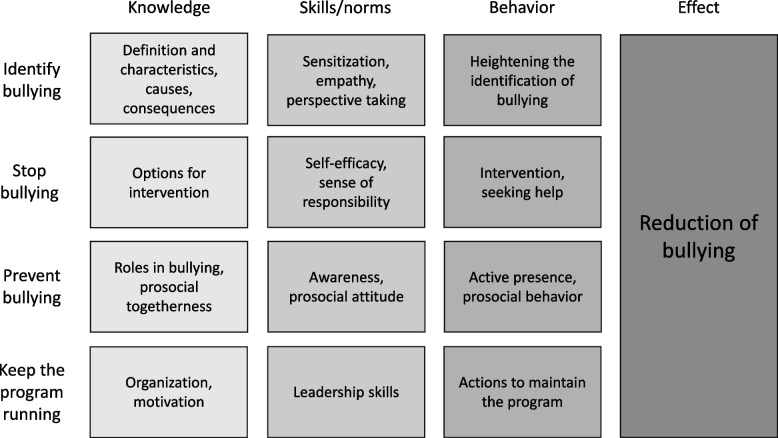
Prevention model: the four secondary goals of Bullying&You assigned to the three determinants of intervention (knowledge, skills/norms, behaviour) necessary for achieving program effect

A prevention model was developed that shows how these goals are to be achieved. The four secondary goals were assigned to the levels of knowledge, skills and norms, behaviour, and finally, effect. Relevant knowledge should be conveyed to all those involved in the program. Building skills and acquiring norms lead to a change in behaviour that ultimately leads to the achievement of the main goal (reduction of bullying) (see Fig. 1). Based on this model, the components of the program were developed and filled with content. Each component of the program refers to at least one point of content in the prevention model.

**Fig. 2:**
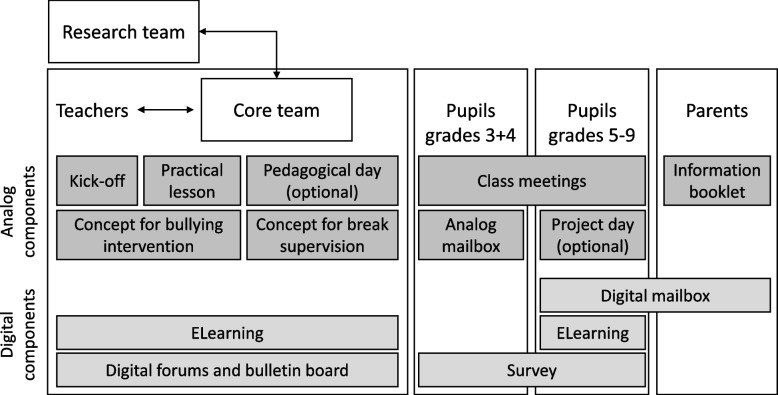
Bullying&You organization model on target groups and program components (analogue and digital)

In *Bullying&You*, analogue program components are combined with digital elements in a blended-intervention approach. An overview of the program components separated by target groups can be found in Fig. 2. Descriptions of the program components are presented in Table 1.

**Table 1:** Analogue and digital components of Bullying&You

Analogue components	Digital components
**Core team** A group of teachers and social workers serves as their school’s steering group. The core team organizes the implementation of the program components and adapts them to the needs of the school. Core team members receive 3 days of training and are supported by the research team	**ELearning** Pupils (grades 5–9) and teachers work on eLearning units in order to build up knowledge and skills in a flexible and self-determined manner
**Kick-off** The program opens with a 1-h talk for the teachers on the topic of bullying. Further, the results of the school’s first survey and the first steps in the program are presented	**Digital mailbox** Pupils (grades 5–9) and parents report experienced or observed bullying incidents to a school-specific email address. The mailbox is looked after by the core team
**Practical lesson** Once per school year, the core team leads a training for the teachers to deepen and practise the intervention concept in small groups using roleplays	**Digital forums and bulletin board** Forums and a bulletin board enable exchange within and across schools
**Pedagogical day** Optionally, the research team organizes a pedagogical day for the school staff on a selection of topics related to bullying	**Survey** Anonymous pupil surveys are carried out regularly to closely monitor bullying and mental health at school
**Concept for bullying intervention** On-the-spot intervention (i.e. all teachers intervene in the event of inappropriate behaviour) leads to follow-up care (i.e. class teachers follow-up on the bullying incident in conversations with the involved pupils)	
**Concept for break supervision** Bullying during breaks is counteracted by optimizing break organization and supervision, creating offers for activities, and restructuring the schoolyard	
**Class meetings** Class teachers lead pre-designed class meetings in which the pupils are made aware of the topic of bullying and acquire options for action. The focus here is on practical experience and exchange	
**Project day** Optionally, class teachers carry out a pre-designed project day on the topic of team building	
**Analogue mailbox** Pupils (grades 3 + 4) use a letterbox at the school to report experienced or observed bullying incidents. The mailbox is looked after by the core team	
**Information booklet** Parents receive a booklet providing important information and tips about bullying and the program	

The eLearning units, forums, the bulletin board, and additional materials are individually delivered via an online access. All teachers and pupils in grades 5 to 9 receive log-in information to an Internet-based platform that requires secured log-in. We decided to use Moodle as our eLearning platform because many German schools already know and use it. Data protection requirements for the Moodle application in the school context were considered. Since the platform is web-based, schools do not need to install specific programs. Users are assigned to different courses depending on their role (pupil, subject teacher, class teacher, core team member, school management) by our research team. The eLearning content was designed as basic Moodle courses for teachers and pupils (first year of the program), advanced Moodle courses for teachers and pupils (further years of the program) as well as additional Moodle courses for special groups of teachers (class teachers, core team members, and school management).

In secondary schools, *Bullying&You* operates in three stages:

1. Preparation (2 months): Principals and core team members become familiar with the program.
2. Implementation (12 months): Pupils work through 6 lessons of the basic course (definition and forms of bullying; individual causes and group mechanisms; prevalence and adverse consequences of bullying; different roles in bullying situations; options for intervention; good togetherness in class). Each lesson consists of an eLearning unit, a short survey, a quiz, and a class meeting. Independent from grade, each pupil gets the same 6 lessons to build up a solid base for the further prevention work. Teachers work on the same 6 topics via eLearning containing target-specific and more detailed information (e.g. the influence of school conditions on bullying development; on-the-spot intervention in 7 steps; guidelines for break supervision and class room management). The core team develops and implements school-specific concepts to reduce bullying and to improve school climate.
3. Continuation (from second program year onwards): Pupils work on age-specific topics in advanced courses, consisting of 4 lessons per school year (grade 6: direct and indirect bullying, grade 7: cyberbullying and gender differences, grade 8: role models and legal aspects, grade 9: prosocial behaviour and helping; grade 5 receives the basic course). Each lesson comprises an eLearning unit, a short survey, and a class meeting. Teachers stay on topic through repetition, peer exchange, pedagogical days, and practical lessons. The core team continuously evaluates and optimizes the anti-bullying concepts of their school.

In primary schools, the structure of the program is somewhat different: Pupils do not receive eLearning. Instead, they get 12 class meetings per school year. These class meetings build on each other and cover 4 major topics: The first topic concerns the creation of a pro-social classroom climate which includes classroom rules, treating others with respect, the importance of diversity, emotion regulation strategies, and teambuilding. The second topic is on the identification of bullying and comprises the definition of (cyber-)bullying, group mechanisms, different roles in bullying situations, and adverse consequences of bullying. The third topic concerns ways to stop bullying as a victim (help seeking, self-assertion), as a bystander, and as a perpetrator. The fourth topic is on creating resources for the transfer to secondary school after grade 4 since bullying is likely to emerge in newly composed groups. It comprises, for instance, strategies to make new friends and to care for oneself. Teachers work through the 6 lessons of the basic course. Afterwards, they stay on topic through repetition, peer exchange, pedagogical days, and practical lessons.

### Criteria for discontinuing or modifying allocated interventions {11b}

Schools participate voluntarily in the program *Bullying&You* and are free to discontinue program implementation at any time. Pupils are free to leave the CRT (research study) at any time, for any reason, without any consequences. From the investigators’ side, there are no general criteria for discontinuing the intervention. Modification of the intervention may occur based on the continuous exchange with and feedback from the core teams regarding barriers that they come across during program implementation. For instance, some schools may not be able to provide the digital requirements for their pupils to complete the eLearning component of *Bullying&You*. In this case, these schools may entirely omit the eLearning for their pupils or the pupils may follow along the eLearning led by their teacher in class instead of completing it by themselves. To track these modifications, program fidelity of each school will be assessed by short teacher surveys at T1 and T2.

### Strategies to improve adherence to interventions {11c}

The systematic program structure helps the schools to plan the next steps and facilitates the implementation. To improve adherence in the IG, the research team meets the core team members of each school regularly (approximately every 2 months) and discusses the implementation of the specific program components. Furthermore, every couple of months, the core teams receive an email summarizing the next relevant steps in the program. Core teams are encouraged to contact the research team if any questions or issues occur. Additionally, the research team monitors the progress of each school’s teachers in completing the 6 eLearning units of the basic course and reports the proportion of completed units to the respective core team once a month. This helps the core team to develop strategies to motivate their teachers in case of low eLearning activity.

### Relevant concomitant care permitted or prohibited during the trial {11d}

Already established unsystematic or single anti-bullying efforts (such as a letter box to anonymously report bullying incidents) are permitted, but other whole-school bullying prevention programs than *Bullying&You* are prohibited during the trial.

### Provisions for post-trial care {30}

We do not anticipate any harms from trial participation. Therefore, there are no provisions for ancillary or post-trial care or for compensations.

### Outcomes {12}

#### First objective

The primary outcome of this trial is the reduction of bullying (prevalence of victims and perpetrators of direct, indirect, and cyberbullying within the last 6 months) assessed by the Bullying Screening [28, 29] from baseline (T0) to two annual follow-ups (T1 and T2) in the IG compared to the CG.

#### Second objective

Secondary outcome is the increase in general health-related quality of life within the last 7 days assessed by the KIDSCREEN-10 [30] in primary schools or KIDSCREEN-27 [31] in secondary schools from T0 to T1 and T2 in the IG compared to the CG.

For secondary schools, additional secondary outcomes are the reduction from T0 to T1 and T2 regarding the following mental health problems:

- Psychological distress within the last 6 months assessed by the Strengths and Difficulties Questionnaire (SDQ; [32])
- Self-harming behaviour within the last 12 and 3 months based on the Self-Injurious Thoughts and Behaviours Interview (SITBI; [33])
- Problematic Internet use within the last 12 months assessed by the Internet Gaming Disorder Scale (IGDS; [34]) and the Social Media Disorder Scale (SMDS; [35])

#### Third objective

To get insight into the effectiveness of specific components of the program, short surveys for teachers will be conducted at T1 and T2. Furthermore, the use of the eLearning platform is recorded continuously (i.e. the proportion of completed eLearning units).

#### Fourth objective

Individual characteristics predicting the program success at the individual level are obtained in secondary schools:

- Personality traits assessed by the Level of Personality Functioning Scale—Brief Form (LPFS-BF; [36])
- Internal self-blame assessed by the Attributions for Victimization Experiences Measure [37, 38]
- Callous-unemotional traits within the last 6 months assessed by the Inventory of Callous-Unemotional Traits—Youth Version (ICU; [39])

### Participant timeline {13}

**Fig. 3:**
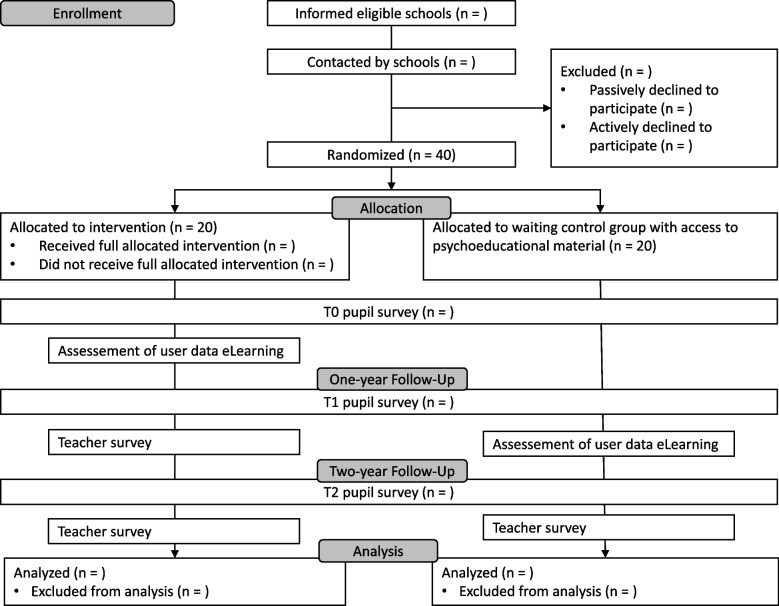
Consolidated Standards of Reporting Trials (CONSORT) 2010 flow diagram

**Fig. 4:**
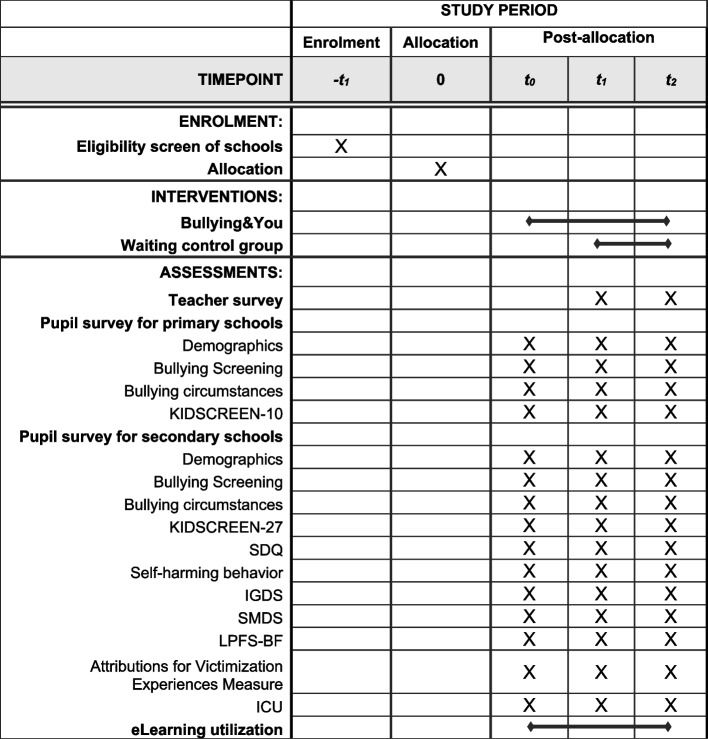
Schedule of enrolment, interventions, and assessments. SDQ, Strengths and Difficulties Questionnaire; IGDS, Internet Gaming Disorder Scale; SMDS, Social Media Disorder Scale; LPFS-BF, Level of Personality Functioning Scale—Brief Form; ICU, Inventory of Callous-Unemotional Traits—Youth Version

The flowchart of the trial is shown in Fig. 3 and the schedule of enrolment, interventions, and assessments in Fig. 4.

### Sample size {14}

To estimate the required sample size, Monte Carlo simulations were carried out using Stata 16.1 with the following assumptions: Two measurement points of 15 secondary schools per group with 10 classes per school and 5 primary schools per group with 4 classes per school are expected to take part in the study. In each class, 25 pupils are expected. Baseline bullying prevalence is estimated to be 15%, and the intervention is expected to lead to a relative reduction in bullying of 25%. The standard deviation (*SD*) of bullying rates between schools is *SD* = 0.2 on the logit scale; the *SD* of classes within schools is *SD* = 0.4 on the logit scale. This leads to an intraclass correlation coefficient on the school level of 0.011 and 0.057 on the class level. The assumptions were chosen so that the simulated data corresponded to the data from the OBPP study [18]. With these assumptions, 1000 data sets were simulated and analysed by three-level mixed logistic regression with group, measurement point, and their interaction as fixed effects and random intercepts on the school (level 3) and class (level 2) level. Using an alpha-error probability of 0.05, with 40 schools and 8500 pupils (4250 IG and 4250 CG) the power would be 86%.

### Recruitment {15}

The recruitment of secondary schools started in September 2021. A complete list of schools in the state of Baden-Wuerttemberg was acquired (public and private schools). For organizational reasons, only schools in two out of four regional councils in Baden-Wuerttemberg that met the eligibility criteria (912 secondary schools) were contacted by email and letter (addressed to principals and social workers). This contact contained our program flyer as well as an invitation to an information event, which took place in October 2021. In January 2022, schools received a reminder of the program by email. The step-by-step decision process of schools regarding their participation included the following: (1) Interested schools receive our detailed information folder, (2) followed by a digital meeting of a member of our research team with the school management and prevention team to clarify questions, and (3) a program presentation by a member of our research team at one of the school’s teacher conferences. In the end, teachers had to vote on the participation of their school in the program. In case of a positive vote, schools signed a participation agreement and were randomized to one of the two experimental conditions. Recruitment of secondary schools was stopped in December 2022 in order to stay within the timeline. Out of the targeted 30 secondary schools, 27 schools were recruited. Since three schools were missing, the number of primary schools was increased by three schools, resulting in a target of 13 primary schools. Thus, the overall target number of schools remained at 40.

Recruitment of primary schools started in September 2022 following an analogue approach. Four hundred and fifty-seven eligible primary schools (principals and social workers) were contacted by email and letter and invited to an information event, which took place in November 2022. Furthermore, 10 primary schools that belonged to a secondary school which already participated in the program were informed about the opportunity to join the program as well. In January 2023, the primary schools received a reminder of the program by email. Inclusion of primary schools was stopped in September 2023.

Recruitment of participants began in June 2022 and will be completed by October 2025.

## Assignment of interventions: allocation

### Sequence generation {16a}

Whole schools were randomly assigned to either IG or CG based on a Randomized Complete Block Design. Even though cluster randomization should only be used when necessary and randomization at the lowest possible level is recommended [40], an alternative randomization strategy was not conceivable due to the whole-school approach underlying our program. Although the program is a complex intervention with components delivered at multiple levels (including individual and class level), substantial components are delivered at the level of the cluster (school). To minimize the disadvantages of this randomization strategy, we followed the guidelines for conducting a cluster randomized trial [40] wherever possible. For example, we allowed for clustering in the sample size calculation as well as in the analysis (see Sect. 14), specified the target of inference (the typical individual) and kept recruiters (class teachers) masked to the cluster allocation (at least at baseline). Furthermore, randomization was automatically performed via a predefined algorithm, stratified by three school types (primary schools = *Grundschule*: grades 1 through 4; A-level schools = *Gymnasium*: comparable to high schools for grades 5 through 12 or 13, more academic, required for enrolment at university; B-level schools = *Realschule/Werkrealschule/Gemeinschaftsschule*: comprises part of general or practical high school education, generally for grades 5 through 9 or 10, allows for the option to commence vocational training, but is insufficient for enrolment at university). This procedure ensured that there was approximately the same number of schools per school type in each group.

### Concealment mechanism {16b}

The allocation sequence was determined by our IT system developer and was implemented in a web application for managing databases (REDCap). One of its features was used to conceal the allocation until a member of the research team assigned a school to its condition by clicking a button for randomization. As this was done by a person other than the system developer, this process ensured that our research group could not influence the allocation of the schools to the two groups. The order in which the schools were randomized depended on the order in which they decided to participate in the program. Randomization took place as soon as the research group received a signed participation agreement from the school. For organizational reasons, randomization took place before baseline. But only principals and core teams were informed about their group affiliation by the research team and were instructed not to disclose their allocation to their teachers or pupils to avoid influences on pupils’ survey responses.

### Implementation {16c}

As mentioned above, the allocation sequence was generated by our IT system developer. Schools were enrolled and assigned to the IG or CG by different members of the research team.

## Assignment of interventions: blinding

### Who will be blinded {17a}

Schools could not be blinded due to the different natures of the IG as opposed to the CG, i.e. immediate as opposed to delayed implementation of a prevention program. Blinding of the researchers is non-applicable, since they are responsible for the program implementation, and data collection relies on online self-reports only. The data analysts will be blinded to group allocation (dummy coded) when conducting the statistical analyses.

### Procedure for unblinding if needed {17b}

Since only data analysts are blinded, unblinding is not needed.

## Data collection and management

### Plans for assessment and collection of outcomes {18a}

Pupils complete anonymous online self-report questionnaires created with LimeSurvey. The responsible teachers read standardized instructions to their pupils and every pupil receives a randomly assigned Login ID (random combination of numbers and letters) for registering on the start page of the survey. In the beginning of the survey, all pupils decide about their participation in the accompanying evaluation study. Approving pupils are asked to create an individual code to enable the association of repeated assessments to the same pupil and receive additional questions during the course of the survey. The assessments take place at school during regular class times and the duration is a maximum of 90 min. The first secondary schools conducted their baseline in March 2022. The first primary schools conducted their baseline in October 2023.

The following scales are used in primary as well as secondary schools:

- Demographics (2 items)
- Bullying Screening ( [28, 29]; 6 items)
- Bullying circumstances for victims, perpetrators, and bystanders based on the Olweus Bullying Questionnaire Revised (OBQ-R; [41]; maximum of 18 items for primary schools and maximum of 25 items for secondary schools, exact number depends on given answers in the Bullying Screening)
- KIDSCREEN-10 ( [30]; 10 items) for primary schools or KIDSCREEN-27 ( [31]; 27 items) for secondary schools:
  o KIDCREEN-10: Cronbach’s alpha = 0.82, test–retest reliability assessed by an intraclass correlation coefficient (*ICC*) = 0.70, and demonstrated validity [30]
  o KIDSCREEN-27: Test–retest reliability *ICC*s = 0.61–0.74 for the different dimensions and was found to be valid [31]

In secondary schools, the following scales are additionally used:

- Strengths and Difficulties Questionnaire (SDQ; [32]; 25 items): Mean Cronbach’s alpha = 0.73 and mean test–retest reliability after 4 to 6 months = 0.62
- Self-harming behaviour within the last 12 and 3 months based on the Self-Injurious Thoughts and Behaviours Interview (SITBI; [33]; 6 items)
- Internet Gaming Disorder Scale (IGDS; [34]; 11 items): Reliability coefficients of 0.82 and 0.83 [42]
- Social Media Disorder Scale (SMDS; [35]; 11 items): Reliability coefficient of 0.57
- Level of Personality Functioning Scale—Brief Form (LPFS-BF; [36]; 12 items): Cronbach’s alpha = 0.82 and promising construct validity

In secondary schools, pupils who agree to participate in the accompanying research also fill in:

- Attributions for Victimization Experiences Measure ( [37, 38]; 17 items): Cronbach’s alpha = 0.71–0.80 for the subscales
- Inventory of Callous-Unemotional Traits—Youth Version (ICU; [39]; 24 items): Cronbach’s alpha = 0.77

All measures are well tested standard instruments and have previously been used in adolescent samples.

In addition to the pupil survey, the evaluation of the program relies on documentation of the actual program components implemented at each school through an annual teacher survey. For this purpose, teachers receive an invitation to an anonymous online survey (T1: only IG; T2: IG and CG), taking a maximum of 5 min to answer. In the questionnaire, all teachers are asked to estimate the workload and the benefit of the program and get the opportunity to give feedback to the program. Class teachers are additionally asked about the amount of eLearning units, class meetings, and project days in their class. Furthermore, the use of the eLearning platform by pupils and teachers is evaluated (i.e. what is the proportion of completed eLearning lessons).

### Plans to promote participant retention and complete follow-up {18b}

Core team members regularly obtain trainings and coordinate and plan the implementation of the program components. This task also comprises the coordination of the baseline and follow-up surveys with their pupils including the coordination of additional assessment dates for previously absent pupils. The research team monitors the progress of the pupil survey and actively contacts the core teams in case of delays or low response rates.

### Data management {19}

Survey data are collected online via LimeSurvey and data on the use of the eLearning platform will be collected online via Moodle. Computerized assessments guarantee the highest level of data integrity and quality, i.e. missing data will be minimized and false data entry will be prevented. Furthermore, online access allows for continuous monitoring of data collection, documentation of access logs, and traceability of all entered data (user and timestamp) as well as restoration of all previous states. A Distributed Replicated Block Device (DRBD)-based cluster provides synchronous replication of all data during data entry on two separate servers and highest availability. The data are stored and processed in a way so that individuals can no longer be identified. No third parties will gain access to the original data. Full and incremental back-ups are conducted following a predefined back-up plan. All research data will be archived for 10 years after the study has ended.

### Confidentiality {27}

Survey and eLearning data will be stored via central servers based in Germany and processed in a way so that individuals cannot be identified. Regarding the pupil survey, the confidentiality of pupils is secured by providing random Login IDs and the creation of individual codes. Data storage and transfer is encrypted. Access to the data is strictly limited to authorized persons and is password-protected. All confidential information is subject to medical confidentiality and to the requirements of the *Landesdatenschutzgesetz Baden-Wuerttemberg, Bundesdatenschutzgesetz*, as well as the European Union General Data Protection Regulation.

### Plans for collection, laboratory evaluation, and storage of biological specimens for genetic or molecular analysis in this trial/future use {33}

No biological specimens will be collected in this trial.

## Statistical methods

### Statistical methods for primary and secondary outcomes {20a}

For the primary outcome (i.e. reduction in victimization and perpetration rates in IG compared to CG; see first objective), three-level mixed effect regressions with the dependent variables bullying victims (yes/no) and perpetrators (yes/no) will be conducted. For secondary outcomes (i.e. reduction in psychological distress, self-harming behaviour, and problematic Internet use in IG compared to CG; see second objective), dependent variables will be scores of the KIDSCREEN-27, SDQ, self-harming behaviour, IGDS, and SMDS (grades 5 to 9) or the scores of the KIDSCREEN-10 (grades 3 and 4). The fixed effects are the factors group (IG, CG), measurement point (T0, T1, T2), and the interaction of both (group × measurement point). The hierarchical random effects are the factors school (level 3) and class (level 2). The analyses are conducted using Wald tests (interaction group × measurement point). In order to investigate the third objective (i.e. effectiveness of specific program components), a variable reflecting the implementation of a specific component of the program (assessed via teacher surveys as well as via eLearning utilization) is included as a covariate and as an interaction term with group, measurement point, and their triple interaction in the model. The triple interaction allows for the identification of the effectiveness of a specific program component. The analysis is repeated for different components. The fourth objective (i.e. individual characteristics predicting program success at the individual level) is investigated by adding a pupil level to the multilevel analysis. The association of surveys from different time points to pupils is ensured by individual codes. Codes with a Levenshtein distance not greater than 2 are assigned to the same pupil. For each individual characteristic (i.e. personality traits, internal self-blame, callous-unemotional traits), the model of objective one is extended by the characteristics at baseline as a predictor and a pupil specific random intercept. In order to compensate for the small number of randomized schools, we will apply a small sample error correction to each analysis using school-level bootstrap. All calculations are performed with a significance level of 0.05.

We orientated our expected effects on the effects of school-bullying prevention programs reported by the latest meta-analyses [14], but would nevertheless consider even smaller program effects as clinically meaningful, first because of the strong causal relationship of bullying experiences with the development of mental disorders [6–9], and second because of the resulting health care costs associated with bullying [43]. Given that this program aims at universal prevention of bullying and associated consequences for mental health, even small effects (i.e. slight changes in mean scores of mental health) can make meaningful contributions to prevalence rates and burden of disease [44].

### Interim analyses {21b}

There are no interim analyses planned. If less than 10 schools would have been recruited within 1 year, the evaluation study must have been stopped due to insufficient sample size and poor attractiveness of the program. However, this number of schools has already been achieved within the first 6 months of recruitment.

### Methods for additional analyses (e.g. subgroup analyses) {20b}

We plan subgroup analyses for gender (boys, girls), school type (primary school, A-level, B-level), and grade groups (grades 3–4, 5–6, 7–9). Furthermore, the influence of school size and the baseline level of bullying victimization on the effectiveness of *Bullying&You* are planned to be analysed.

### Methods in analysis to handle protocol non-adherence and any statistical methods to handle missing data {20c}

The analysis is an intention to treat analysis; whole schools were randomly assigned to the two conditions intervention group vs. control group. In order to enable sub-analyses differentiated by program dose, a score based on the intensity of the central program components will be created for each school.

Sensitivity analyses are done on the complete-case sample, i.e. excluding schools where measurement points are missing.

To deal with our fourth objective (individual characteristics predicting the program success at the individual level), the association of questionnaires from different time points to pupils is required. Therefore, individual codes with a Levenshtein distance not greater than 2 are assigned to the same pupil. Apart from this strategy to reduce missings, no missing data is imputed.

### Plans to give access to the full protocol, participant-level data, and statistical code {31c}

The dataset and statistical code can be made available by the corresponding author upon reasonable request.

## Oversight and monitoring

### Composition of the coordinating centre and trial steering committee {5d}

This trial is designed, performed, and coordinated by the *Bullying&You* research team at the Child and Adolescent Psychiatry Department at the University Hospital Heidelberg. Day to day support for the trial is provided by the principle investigator, the study coordinator, and the research assistants. The entire team meets approximately once a month. Additional support is provided by an IT system developer and a data analyst.

### Composition of the data monitoring committee, its role and reporting structure {21a}

We have not established a Data Monitoring Committee (DMC). However, despite the absence of a DMC, we are committed to ensuring scientific and ethical standards. We implement alternative monitoring measures, such as regular reviews of trial progress and monitoring data safety. Additionally, we maintain close collaboration with regulatory authorities and the ethics committee.

### Adverse event reporting and harms {22}

There are no obvious harms for participants of the trial and it has been proven that addressing self-harm behaviour to children and adolescents does not have an iatrogenic effect, but can even have a positive preventive effect—especially in younger participants [45]. Nevertheless, completing the survey could potentially lead to psychological distress for those pupils affected by bullying. In order to cope any strain that may arise, every school appoints a qualified contact person (e.g. social worker, counsellor, or core team member) whom the pupils can consult for support. This contact person is communicated to the pupils at the beginning of the survey. Any serious adverse effects of the study can also be reported to the research team. Furthermore, to prevent harms, everybody can deny participation in the survey as part of the program and/or in the accompanying study at any time without stating the reason and without any individual disadvantage for subsequent school career or medical care.

### Frequency and plans for auditing trial conduct {23}

We do not have a specific audit plan for this trial. However, we acknowledge the importance of monitoring and ensuring the integrity of the trial.

### Plans for communicating important protocol amendments to relevant parties (e.g. trial participants, ethical committees) {25}

The study was appraised and approved by the Ethics Committee of the Medical Faculty at the University of Heidelberg (S-471/2020), as well as of the Ministry of Education of the state of Baden-Wuerttemberg. Furthermore, the study was registered at a WHO trial registry (German Clinical Trials Register, DRKS00028183). In case of relevant protocol modifications, the institutional review board of the Medical Faculty at the University of Heidelberg is informed and an amendment is submitted. Furthermore, information within the German Clinical Trials Register is updated to inform the public about possible changes. In case any amendments concern the participants, they are also informed about the changes.

### Dissemination plans {31a}

To disseminate the results within the scientific community, research publications in peer-reviewed journals and conference contributions are planned. Access to the protocol is ensured through the registration and regular update of the trial in the German Clinical Trials Register. The 40 pilot schools will receive the results of our evaluation study via digital information and events. Depending on the results, information about the program, its effectiveness, and availability (after-study stage) shall be provided to a larger scale of schools, teachers, and other professionals working in the field. Awareness in the general public will be increased by the *Bullying&You* website (www.mobbing-und-du.de) and press campaigns.

## Discussion

Peer relationships play an important role in children’s and adolescents’ lives, and the importance of peer relationships steadily rises with increasing age. Bullying is a man-made, intentional aggression, which is consistent with the defining features of maltreatment or abuse. As a form of peer‑to‑peer maltreatment, bullying implies severe consequences for mental health and academic achievement, mostly for victims [5]. While there is evidence for the overall effectiveness of school-based bullying prevention programs [14], randomized trials are still urgently needed, and further developments of feasible as well as permanently effective programs are critical. There is still a lack of systematic bullying prevention and, therefore, there exists the need for the development of new, innovative research-based programs in Europe to fill this gap [26]. Currently, programs are often only translated or adapted and do not correspond to the needs of the local school system and culture. However, newly developed interventions must be evaluated and, in a second step, improved based on the obtained results. Modern and flexible program components like eLearning units are needed to make anti-bullying programs more feasible and attractive to the pupils and school staff. Additionally, the school staff’s perspective should be included in the development of the program by consulting focus groups and programs should also target primary schools, as the effect of anti-bullying programs proved to be larger for younger children [46].

Therefore, the Child and Adolescent Psychiatry Department at the University Hospital Heidelberg has developed the program *Bullying&You*. This school-based prevention program aims to reduce bullying by teaching pupils and teachers how to identify, stop, and prevent bullying. Using a CRT design on a large-scale sample, the aim of this trial is to investigate the effectiveness of the *Bullying&You* program at 30 secondary and 10 primary schools.

Despite the well-designed trial and the promising anti-bullying intervention, we may also face some barriers. First, some schools might not be able to profit from the various advantages of the digital program components (e.g. eLearning) because they lack the required digital infrastructure. At times, teachers in Germany are also still rather sceptical or feel insecure when dealing with digital media. These challenges can be addressed by offering modifications of the relevant program components, as well as by providing guidelines for, and intuitive designs of, the digital media. Second, interrelations of aspects affecting the program effectiveness are very complex: some program components might only work for certain age groups, certain forms of bullying, or a particular gender. Further, a program component may not be effective on its own, but in combination with others, proves to be successful. Mediators and moderators of preventive programs have scarcely been researched (‘*What works, for whom, and under what circumstances*’, [47], p. 438). Due to this complexity, we will need to analyse our data carefully to draw our conclusions. Third, implementing a whole-school approach may take some time. Thus, changes in bullying and mental health might not yet be significant after only 1 or 2 years of program implementation. As our evaluation study only comprises 3 measurement points (baseline T0; follow-up + 12 months T1; follow-up + 24 months T2), additional follow-up time points are highly desirable to evaluate the long-term effectiveness and sustainability of *Bullying&You*.

### Implications and future impact

As early as more than 10 years ago, Spiel and Strohmeier [48] expressed their growing disappointment about the slow and incomplete application of research results in the field of education. Results of developmental and educational psychology are overlooked due to a lack of structures and resources, clear standards of evidence, failing transfer into practice, and an existing resistance to change. Intensive cooperation between researchers, politicians, administrators, and the media is required in order to change this unsatisfactory situation for the better. We want to contribute to this change by developing an innovative and feasible bullying prevention program based on state-of-the-art scientific findings. The current mental health crisis in children and adolescents [11], the advantages of the school setting for preventive efforts [12], as well as the lack of German-language school-based bullying prevention programs with proven effectiveness [13] clearly increase the need for such a program.

To conclude, bullying deserves a high level of attention and efforts to be successfully identified, stopped, and prevented. Pupils and teachers must acquire knowledge and build up skills to change their behaviour. If the whole school community feels responsible, actively intervenes in bullying situations, and enforces prosocial behaviour, bullying can indeed be reduced in the school setting and beyond (e.g. in the cyberspace). Unfortunately, available school-based anti-bullying programs are limited in their application; systematic bullying prevention is still lacking within the German school system. *Bullying&You* may help to overcome existing barriers and support schools in becoming a safe place for all of their pupils.

## Trial status

Recruitment of schools started in September 2021 and was stopped in September 2023. Recruitment of participants began in June 2022 and will be completed by October 2025. The current protocol is version 1 of 2 March 2020. The trial is scheduled to be completed by October 2025.

## Data Availability

The research team and the data analyst will have access to the final trial dataset.

## Abbreviations

CG: Control group
CRT: Cluster randomized trial
DHI: Digital health intervention
ICC: Intraclass correlation coefficient
ICT: Information and Communication Technology
ICU: Inventory of Callous-Unemotional Traits
IG: Intervention group
IGDS: Internet Gaming Disorder Scale
LPFS-BF: Level of Personality Functioning Scale—Brief Form
OBPP: Olweus Bullying Prevention Program
OBQ-R: Olweus Bullying Questionnaire Revised
SD: Standard deviation
SDQ: Strengths and Difficulties Questionnaire
SITBI: Self-Injurious Thoughts and Behaviours Interview
SMDS: Social Media Disorder Scale
WHO: World Health Organization
